# Manganese reductive dissolution coupled to Sb mobilization in contaminated shooting range soil

**DOI:** 10.1007/s00253-024-13133-2

**Published:** 2024-04-10

**Authors:** Lara Costa, Mathieu Martinez, Marcel Suleiman, Rolf Keiser, Moritz Lehmann, Markus Lenz

**Affiliations:** 1 Institute for Ecopreneurship, School of Life Science, University of Applied Sciences and Arts Northwestern Switzerland (FHNW), Hofackerstrasse 30, 4132 Muttenz, Switzerland; 2 Department of Environmental Science, University of Basel, Bernoullistrasse 30, 4056 Basel, Switzerland; 3 ARMASUISSE Competence Center Soil, Guisanplatz 1, 3003 Bern, Switzerland; 4 Sub-Department of Environmental Technology, Wageningen University, 6700 EV Wageningen, The Netherlands

**Keywords:** Antimony mobility, Antimony environmental fate, Trace metal fate, Metalloid risk assessment, Redox-stat bioreactor

## Abstract

**Abstract:**

A “redox-stat” R_MnR_ bioreactor was employed to simulate moderately reducing conditions (+ 420 mV) in Sb-contaminated shooting range soils for approximately 3 months, thermodynamically favoring Mn(IV) reduction. The impact of moderately reducing conditions on elemental mobilization (Mn, Sb, Fe) and speciation [Sb(III) versus Sb(V); Fe^2+^/Fe^3+^] was compared to a control bioreactor R_CTRL_ without a fixed redox potential. In both bioreactors, reducing conditions were accompanied by an increase in effluent Sb(V) and Mn(II) concentrations, suggesting that Sb(V) was released through microbial reduction of Mn oxyhydroxide minerals. This was underlined by multiple linear regression analysis showing a significant (*p* < 0.05) relationship between Mn and Sb effluent concentrations. Mn concentration was the sole variable exhibiting a statistically significant effect on Sb in R_MnR_, while under the more reducing conditions in R_CTRL_, pH and redox potential were also significant. Analysis of the bacterial community composition revealed an increase in the genera *Azoarcus*, *Flavisolibacter*, *Luteimonas*, and *Mesorhizobium* concerning the initial soil, some of which are possible key players in the process of Sb mobilization. The overall amount of Sb released in the R_MnR_ (10.40%) was virtually the same as in the R_CTRL_ (10.37%), which underlines a subordinate role of anoxic processes, such as Fe-reductive dissolution, in Sb mobilization. This research underscores the central role of relatively low concentrations of Mn oxyhydroxides in influencing the fate of trace elements. Our study also demonstrates that bioreactors operated as redox-stats represent versatile tools that allow quantifying the contribution of specific mechanisms determining the fate of trace elements in contaminated soils.

**Key points:**

• *“Redox-stat” reactors elucidate Sb mobilization mechanisms*

• *Mn oxyhydroxides microbial reductive dissolution has a major role in Sb mobilization in soils under moderately reducing conditions*

• *Despite aging the soil exhibited significant Sb mobilization potential, emphasizing persistent environmental effects*

**Supplementary Information:**

The online version contains supplementary material available at 10.1007/s00253-024-13133-2.

## Introduction

In Switzerland, approximately 4000 shooting ranges are comprised in the register of polluted sites and account for 200 tones of Pb annually entering Swiss soils (Bundesamt für Umwelt BAFU 2020). Pb bullets contain 2 to 5% of metallic Sb as a hardener. Sb is classified as a priority pollutant by both the US Environmental Protection Agency (USEPA) and the Council of the European Union (EU) (Filella et al. 2002a, b; Bagherifam et al. 2019). The maximum admissible value for Sb concentrations in drinking water, according to the World Health Organization (WHO), is 0.05 mg L^−1^, and the benchmark value is set at 0.02 mg L^−1^ (WHO 2018).

In an aqueous solution, at neutral pH, Sb(III) and Sb(V) do not persist in free ionic form, but rather undergo hydrolysis to form Sb(OH)_3_^0^ (antimonite) and Sb(OH)_6_^−^ (antimonate), respectively. Sb(V) is more stable under oxidizing conditions, while Sb(III) predominates under reducing conditions (Filella et al. 2002a, b). However, both these species have been observed to occur outside their predicted thermodynamic stability ranges in natural fresh waters.

The weathering and corrosion of spent bullets in oxic conditions result in the mobilization of Pb in cationic form (Pb^2+^) and of Sb in anionic form (Sb(OH)_6_^−^, Sb(V)), imposing a risk of entering the food chain. The biotic and abiotic mechanisms controlling Sb leaching from soils into ground and surface waters, however, are not yet fully understood. During periods of waterlogging, soils can become anoxic, potentially fostering the reductive dissolution of Sb-hosting phases and resulting in the release (mobilization) of sorbed Sb(III) and Sb(V). For instance, Sb(III) strongly adsorbs onto Fe minerals, such as goethite, over a pH range of 3 to 12, whereas Sb(V) preferentially adsorbs at neutral pH (Leuz et al. 2006a; Liu et al. 2015). Low pH also favors the adsorption of Sb(III) and Sb(V) onto Mn minerals, such as pyrolusite and manganite, with the rate of adsorption decreasing with increasing pH (Wang et al. 2021). Batch mesocosm experiments have suggested that the transition to reducing conditions at first leads to the immobilization of Sb by its reduction to Sb(III) due to microbial respiration/activity. Sb(III) then sorbs to Fe phases and can subsequently be released when even more strongly reducing conditions cause the dissolution of the latter (Hockmann et al. 2014a, b). In strongly reducing soils, Sb mobility is therefore controlled by the interplay and balance between multiple (reductive) reactions, e.g., the reduction of Sb(V) to Sb(III), the sorption of Sb(III) to and the release from Mn and Fe oxyhydroxides upon their reductive dissolution (Filella et al. 2009; Wilson et al. 2010; Hockmann et al. 2014a, b).

In most soils, waterlogging is only temporary, and the redox conditions are less extreme, fluctuating between oxidized and moderately reducing conditions. Furthermore, within soil microaggregates, slightly reducing conditions may occur (Lacroix et al. 2023). These moderate redox potentials thermodynamically favor only some of all possible reductive reactions. For instance, column experiments have demonstrated that in soils that undergo moderately reducing conditions (approx. *E*_h_ = 300 mV), microbially catalyzed Mn(IV) reduction dominates the release of Sb in form of Sb(V) (Hockmann et al. 2014a, b). Due to the availability of a variety of possible electron acceptors, the redox potential often decreases rapidly upon waterlogging (Hockmann et al. 2014a, b), making it difficult to study Sb release under transient, moderately reducing conditions.

Sb mobility is modulated by the presence of specific bacteria that can affect Sb bioavailability and speciation, either directly by Sb dissimilatory reduction and/or indirectly through Mn and Fe dissimilatory reduction. In this study we used a bioreactor with redox potential control (a “redox-stat”) (Rajpert et al. 2018) to investigate biotic and abiotic constraints on Sb mobilization specifically under moderately reducing conditions. In particular, the involvement of Mn reductive dissolution in Sb mobilization was studied by controlling the redox potential in the bioreactor (referred to “R_MnR_”) at moderately reducing conditions (minimum of 350 mV) that thermodynamically favor only the reduction of Mn oxyhydroxides (and suppress other redox reactions that may occur under more reducing conditions, such as Fe hydr(oxides) reduction, and Sb reduction). Element (Sb, Mn, Fe) mobilization from the soil to the water was quantify throughout the experiment, as well as speciation of Sb. A sequential extraction scheme was used to determine metal fractionation. Element mobilization in R_MnR_ was then compared to a bioreactor not operated as a redox-stat, serving as control (R_CTRL_). The soil microbial community was characterized using next-generation metagenomic sequencing to gain a deeper understanding of the key microbial genera that may be involved in Mn reduction–associated Sb mobilization under moderately reducing soil conditions.

## Material and methods

### Soil sampling and characterization

Soil was collected in the surroundings of the backstop berm of a military shooting range located in Brocheten, Switzerland (47° 19′ 5218″ N and 007° 34′ 6546″ E; canton Solothurn). The soil originated from a longer-term (> 30 years) contaminated shooting range soil characterized by nutrient-poor, moderately dry pasture (BSB + Partner, Ingenieure und Planer 2007).

Hotspots of Sb and Pb concentration were determined using a handheld XRF device upon collection. There, soil was taken from the surface layers (0–30 cm) and, after roots and sods were removed, was homogenized, air-dried (< 2 wt% water content), passed through a sieve (≤ 2 mm), and stored in buckets in the dark. The soil texture qualified as coarse sand (7.60% humus, 27.10% pebbles, 27.30% silt) according to the international system for particle size distribution analysis and contained 16.48 ± 0.31% CaCO_3_ and 4.41% organic carbon. Soil pH was 7.40 ± 0.01 (measured in 0.01M CaCl_2_). It displayed a cation exchange capacity of 0.34 ± 6.72 meq/100 g soil. The total metalloid concentrations (Mn, Fe, Sb, and Pb) in the soil samples collected were quantified by energy-dispersive X-ray fluorescence spectroscopy (SPECTRO XEPOS, AMETEK, Germany). The mean concentrations in the soil used in this study were 1255 ± 49 mg kg^−1^ Mn, 42,018 ± 535 mg kg^−1^ Fe, 27 ± 4 mg kg^−1^ Sb, and 766 ± 84 mg kg^−1^ Pb. The certified reference material “BCR-176R fly ash” (European Community Bureau of Reference, BCR, Sigma-Aldrich, Switzerland) was used for calibrating XRF analyses, yielding 92% of the certified expected elemental recovery (Table S1).

In order to determine metal partitioning/distribution in the soil before and after treatment in the reactors (i.e., to constrain how metals are bound or associated with different soil phases), a four-step sequential extraction/digestion on certified reference material “BCR-701” was used according to BCR recommendations (European Comission 2012), followed by ICP-MS analysis of the single extracted fractions. More precisely, metals were extracted by four consecutive steps into the following fractions (F1-F4): “exchangeable” F1 (step 1 with 0.11 M acetic acid at pH 2), “reducible” F2 (step 2 with 0.5 M hydroxylammonium chloride at pH 2), “oxidizable” F3 (step 3 with 8.8 M hydrogen peroxide at pH 2), and “residual” F4 (step 4 with aqua regia). The non-extractable fraction of Sb was calculated by difference between the total Sb content (determined by XRF) and Sb extracted in F1 to F4.

### Thermodynamic modeling

The soil–water system of the bioreactors was simulated using thermodynamic equilibrium modeling with Visual MINTEQ database (Gustafsson 2014) implemented in Geochemist Workbench version 12.0 (Champaign, Illinois). The full model containing all species in the database, as well as a reduced model (suppressing different Sb oxides), is presented in the Supplementary information (Figure S1a, Figure S1b). The computed Pourbaix diagrams indicate that, at neutral pH, Sb(V) reduction to Sb(OH)_3_ would occur at a redox potential lower than *E*_h_ =  ~ 125 mV.

Regarding Mn (Figure S1c), the diagram suggested that at *E*_h_ < 450 mV, the conversion of Mn(III) (as BixByte) to dissolved Mn2 + would be expected.

To safely suppress both Sb and Fe reduction (at <  − 250 mV, Figure S1d), a minimal redox potential of *E*_h_ =  ~ 400 mV was chosen for the redox-stat. The range of 450 mV < *E*_h_ < 350 mV is hereafter referred to as “manganese-reducing conditions.”

### Bioreactor operation

The experiments were performed in two 1-L continuously stirred-tank bioreactors (CSTR, Multifors, INFORS HT, Bottmingen, Switzerland) in mesophilic (21 ± 5 °C) conditions at a pH of 7.0 ± 0.3 (Figure S2). Soils were added at a solid–liquid ratio of 10% (w/v), and the slurry was stirred at 70 rpm. The hydraulic retention time was set to 48 h, where the bioreactor was fed with an anoxic artificial rainwater solution with the following composition (in g L^−1^): 2.13 NaCl, 3.63 MgCl_2_·7H_2_O, 3.06 CaCl_2_·6H_2_O, 0.37 KCl, 1.53 NaNO_3_, and 2.98 Na_2_SO_4_. The headspace of the bioreactors was constantly flushed with N_2_ during operation. The bioreactors were covered with aluminum foil to protect them from light and thus to prevent photooxidation, as well as algae growth. The principal aim of this study was to acquire a comprehensive understanding of the distinct contribution of Mn reduction in Sb mobilization. To achieve this, in one bioreactor (R_MnR_), the redox potential was set to “manganese-reducing conditions” (see the “Thermodynamic modeling” section), as previously described (Rajpert et al. 2018). In R_MnR_, the redox potential was stabilized by sporadic air injections into the reactor headspace. As an adaption to the setup used by (Rajpert et al. 2018), instead of a standard digital redox—processor, a NI CompactRIO and LabVIEW (National Instruments, Ennetbaden, Switzerland) was used to control the gas valves (compressed air input), which allowed us also to record *E*_h_ data in 30-min intervals instead of manual readouts. In the other bioreactor, which served as a control (R_CTRL_), we let the redox conditions develop naturally, allowing for the establishment of much more reducing conditions in the incubated soil.

### Liquid phase analysis

Samples (15 mL) were collected in duplicates from the reactors through a sterile needle and syringe and ultra-centrifuged (Amicon Ultra-4 Centrifugal Filter Unit, 30 kDa MWCO; 4500 rcf, 21 °C, 15 min). The concentrations of total dissolved Sb, Pb, Mn, and Fe were quantified using an Agilent 8800 qqq-ICP-MS (Agilent Technologies AG, Basel). Prior to analysis, the samples were diluted (1:200) in 3% HNO_3_ (Merck, Switzerland). All elements were measured in helium-collision mode, monitoring masses ^55^Mn, ^56^Fe, and ^121^Sb. The limits of quantification (LOQ) were determined as ten times the standard deviation (*σ*) of a set of blanks (*n* = 10), while the limit of detection (LOD) was determined as three times *σ*. The determined LOQ values for Sb, Mn, and Fe are 0.69 µg L^−1^, 0.35 µg L^−1^, and 1.19 µg L^−1^, respectively. Correspondingly, the LOD values for Sb, Mn, and Fe are 0.21 µg L^−1^, 0.10 µg L^−1^, and 0.36 µg L^−1^, respectively.

Antimony speciation analyses were carried out by LC-ICP-MS (Lintschinger et al. 1998) (Agilent Technologies AG, Basel, 1260 pump series). The samples for Sb speciation were handled in the glove box, filtered through 0.45-mm PVDF filters, stabilized in the degassed mobile phase (10 mM Na-EDTA, 1 mM phthalic acid, and 2% methanol at pH 4.5), stored at 4 °C, and measured within 72 h after sampling. An anionic exchange column (Hamilton PRP-X100, 250 × 4 mm, 10 µm) with an isocratic elution was used for the separation. The injection volume was 100 µL, and the flow rate was 0.8 mL min^−1^. To improve nebulization, the flow rate was split in half before entering the nebulizer of the ICP-MS. The calibration standards of Sb(III) and Sb(V) were freshly prepared from Sb_2_O_3_ dissolved in 2M HCl (Merck, Switzerland) and from KSb(OH)_6_ (Merck, Switzerland) dissolved in water, respectively. The dissolved reduced iron (Fe^2+^) concentration was measured spectrophotometrically using 1,10-phenanthroline (Fadrus and Malý 1975). From the measured trace metal concentration changes in the reactor, trace metal mobilization rates were calculated as described in Equation S3 and Equation S4. Dissolved organic carbon (DOC) was quantified using a total organic carbon analyzer (TOC-L Shimadzu).

### Soil DNA extraction and metagenomic sequencing

Microbial genomic DNA was extracted from the soil samples using the DNeasy PowerSoil Pro Kit (Qiagen, Netherlands). DNA was quantified using a Qubit™ dsDNA HS Assay Kit (Thermo Fischer Scientific, Waltham, MA, USA). Gene-targeted sequencing of the 16S ribosomal RNA was performed using the Quick-16S™ NGS Library Preparation Kit (Zymo Research, Irvine, CA). The 515F-806R primers were used as they were designed to amplify prokaryotes (Bacteria and Archaea) and target the V3-V4 region of the 16S rRNA gene. The sequencing library was prepared in real-time PCR machines to control the cycles and prevent PCR chimera formation. The final PCR products were quantified with qPCR fluorescence and pooled together based on equal molarity. The final pooled library was cleaned with Select-a-Size DNA Clean & Concentrator™ (Zymo Research, Irvine, CA) and quantified in Qubit. The final library was sequenced on Illumina® MiSeq™ with a V3-V4 reagent kit. Raw sequencing reads were processed in R using dada2 (Callahan et al. 2016), including the removal of primer sequences, quality control, estimation of error rates, and detection and removal of chimeras. The resulting sequence table was aligned to version 138 of the SILVA ribosomal RNA database (Quast et al. 2013), specifically the non-redundant dataset 99. Subsequently, a phyloseq object was constructed using the phyloseq package (McMurdie and Holmes 2013), which encompassed an amplicon sequence variant (ASV) table, a taxonomy table, and sample data. Further analysis was conducted using the R packages phyloseq (McMurdie and Holmes 2013) and vegan (Oksanen et al. 2020). Raw sequencing data are deposited on NCBI SRA archive under bioprocess accession number PRJNA997570.

### Regression analysis

A multiple linear regression to a significance level of *p* < 0.05 was employed to determine possible correlations between Sb total in the effluents and Fe, Mn, Pb, and DOC concentrations, as well as the redox potential and pH for the entire experiment (see Table S4).

## Results

### Elemental mobilization

**Fig. 1:**
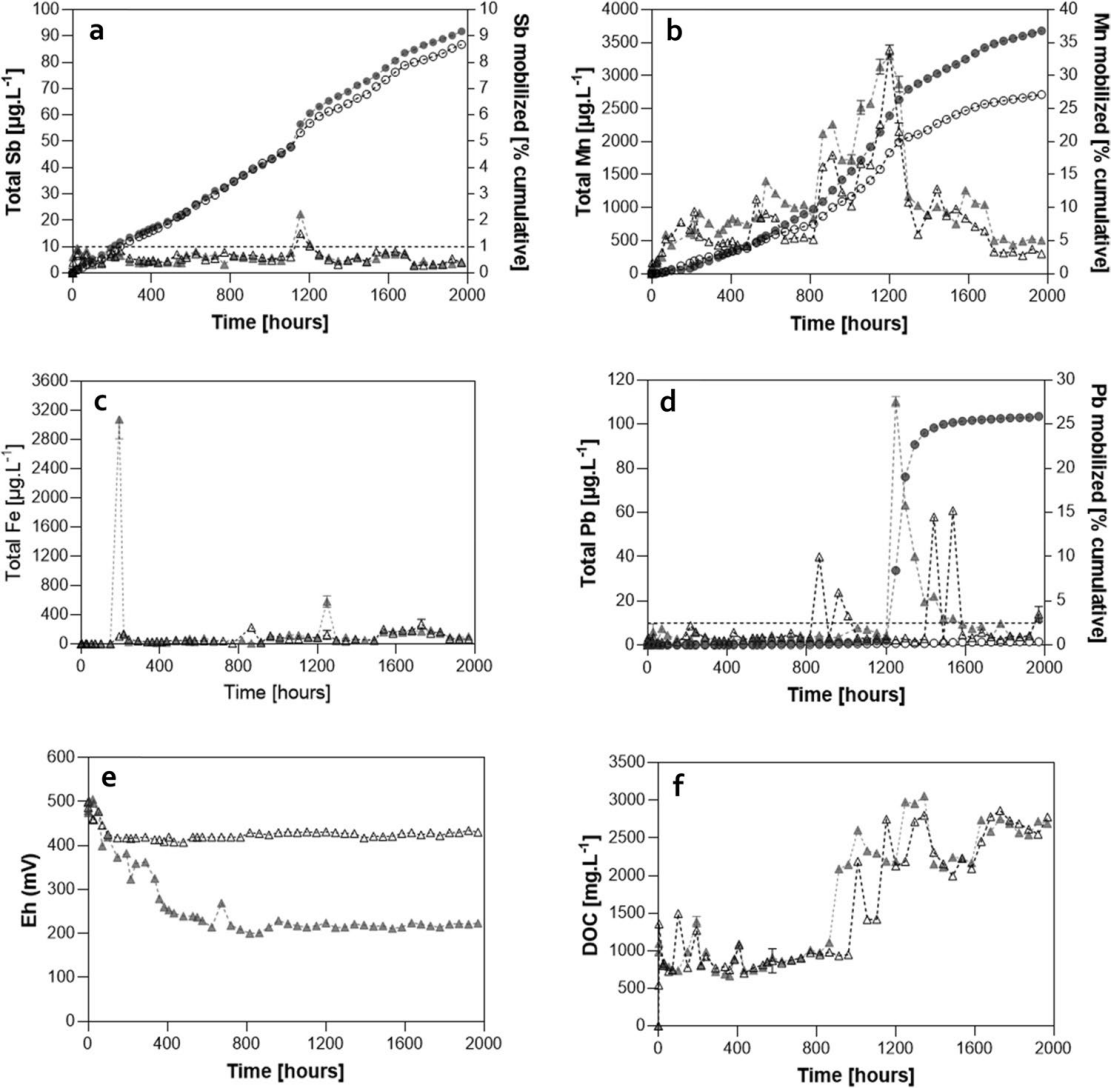
Mobilization of Sb (**a**), Mn (**b**), Fe (**c**), Pb (**d**), redox potential (**e**), and DOC (**f**) in R_MnR_ (open symbols) and R_CTRL_ (closed symbols). Elemental mobilization is indicated as concentration in the effluent [μg L^*−*^.^1^] (triangles; on primary *y*-axis in **a**–**d**) and as cumulative element mobilized [% of initial] (circles; secondary axis in **a**–**d**). The dashed horizontal lines in **a** and **d** represent the most stringent drinking water limit values for Sb and Pb, respectively (details see text)

The Sb concentration in R_MnR_ and R_CTRL_ was typically below 10 µg L^−1^, yet a transient peak in concentration was observed: 15.0 ± 0.1 µg L^−1^ in R_MnR_; 22.4 ± 0.1 µg L^−1^ in R_CTRL_ at 1153.5 h (Fig. 1a). These relatively low fluctuations in the Sb effluent concentrations resulted in a steady increase of Sb mobilized cumulatively from the soil in both R_MnR_ and R_CTRL_, reaching 10.4% Sb (2.7 ± 0.0 mg kg^−1^ soil) and 10.4% (2.8 ± 0.2 mg kg^−1^ soil) at the end of reactor operation (Fig. 1a), respectively. XRF analysis of the remaining solids revealed a decrease of the Sb concentration from 27 ± 4 to 24 ± 3 mg kg^−1^ Sb (R_MnR_), and to 23 ± 3 mg kg^−1^ Sb (R_CTRL_), upon termination of the bioreactor operation (Table S3).

R_MnR_ and R_CTRL_ also showed a steady increase in dissolved Mn(II) mobilized until 900 h. Thereafter, the Mn mobilization rate increased considerably until around 1200 h, before it decreased again until the end of the incubation. The cumulative fraction of Mn mobilized calculated via effluent concentrations was 27.9% (356 ± 13 mg kg^−1^) in R_MnR_ and 37.5% (481 ± 11 mg kg^−1^) for R_CTRL_ (Fig. 1b), respectively. The Mn concentration in solids decreased from 1255 ± 50 mg kg^−1^ in the starting soil material to 992 ± 13 mg kg^−1^ (R_MnR_) and 814 ± 5 mg kg^−1^ (R_CTRL_) in the residual soil after reactor operation (Table S3).

By contrast, Fe showed little mobilization in neither of the reactors (Fig. 1c) until the end of operation (0.17% and 0.09% cumulatively in R_MnR_ and R_CTRL_, respectively), which corresponded to an insignificant decrease in solid Fe concentration in the soil (from 42,018 ± 535 to 40,850 ± 802 mg kg^−1^ in R_MnR_ and 40,507 ± 163 mg kg^−1^ in R_CTRL_) (Table S3). The mobilization of Pb (Fig. 1d) was also generally low (average ~ 6 µg L^−1^ and 10 µg L^−1^ in R_MnR_ and R_CTRL_, respectively), with the exception of three sampling events with increased Pb concentrations in R_CTRL_ (40–110 µg L^−1^). These sporadic mobilization pulses contributed the bulk to the cumulative Pb mobilized in R_CTRL_ (total of ~ 25%). DOC effluent concentrations were similar in both reactors during the experiment (Fig. 1f).

**Fig. 2:**
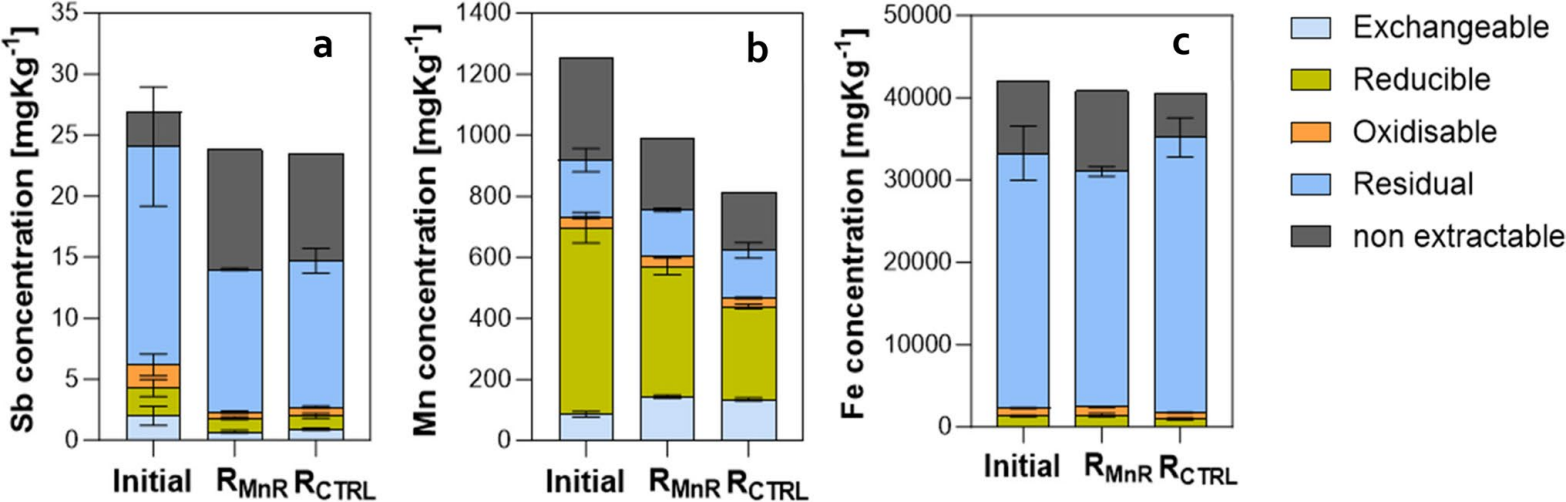
Metal fractionation of Sb (**a**), Mn (**b**), and Fe (**c**) in the initial soil and the soils after treatment in R_MnR_ and R_CTRL_ (for details refer to text)

**Fig. 3:**
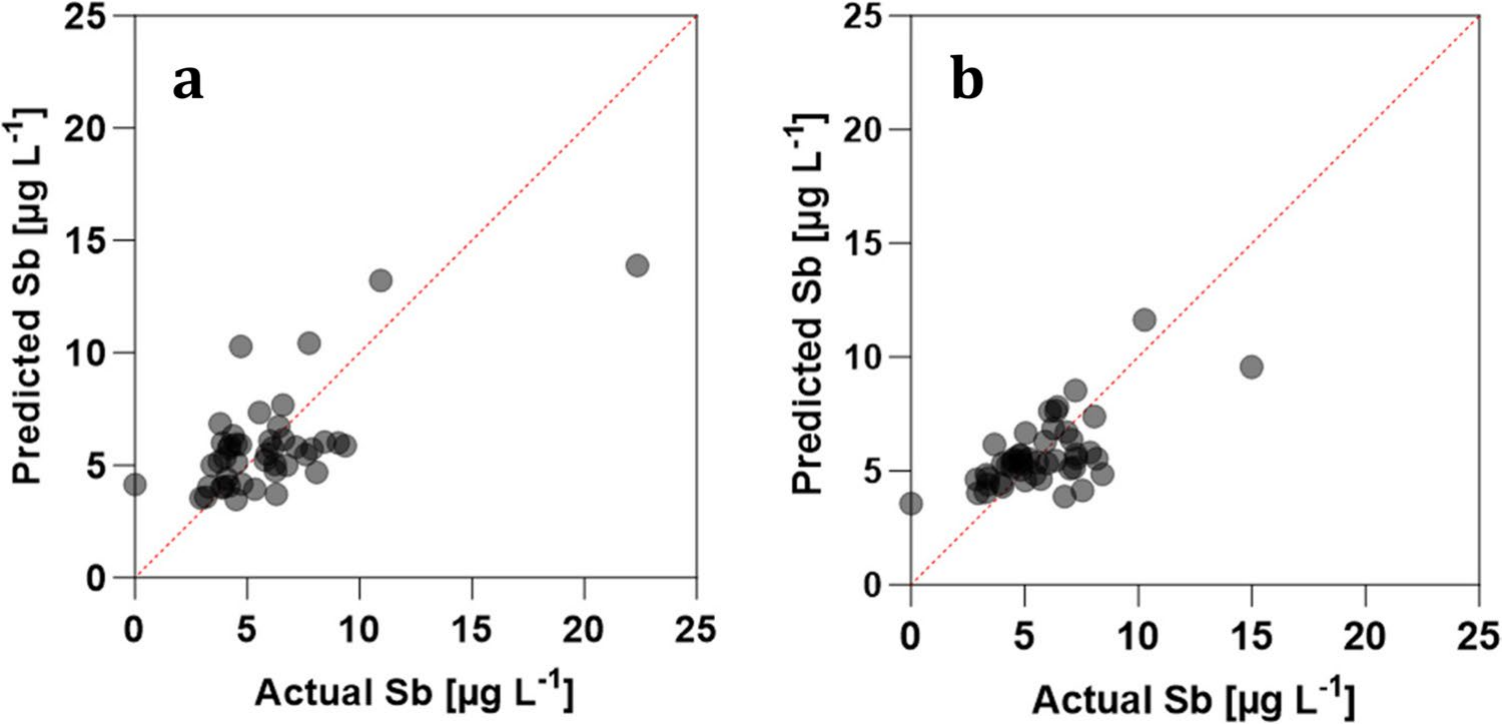
Actual versus Sb effluent concentrations [µg/L] predicted by multiple linear regression in R_MnR_ (**a**) and R_CTRL_ (**b**) (for details refer to text and supplementary materials 1.3)

The multiple linear regression analysis revealed Mn as the most significant independent variable affecting Sb mobilization (*p* = 5 × 10^−5^ for R_MnR_ and 1 × 10^−6^ for R_CTRL_, resp.). While in R_MnR_ it was the only significant independent variable affecting Sb, under the lower redox conditions in R_CTRL_, redox potential and pH had a significant impact as well (Table S4). In contrast, Fe, Pb, and DOM effluent concentrations did not significantly correlate with Sb concentrations in the bioreactor. Overall, in both reactors, Mn was an excellent predictor of Sb effluent concentrations (Figs. 2 and 3).

### Redox potential, Fe, and Sb speciation

The *E*_h_ was controlled in R_MnR_ for the whole experimental period (> 3 months) (Fig. 1e). After an initial stabilization period of 72 h, the redox remained constant in R_MnR_ at *E*_h_ = 422 ± 7 mV, which is conducive to Mn(IV) reduction. For R_CTRL_, the *E*_h_ decreased to < 265 mV within 625 h of operation and remained at around 200 mV, thereafter, until the end of the operation. The pH of the soil solution remained mostly neutral during operation in both reactors (both 6.99 ± 0.15) (data not shown). Dissolved Fe(II) was detected exclusively in R_CTRL_ on few occasions, with concentrations < 200 µg L^−1^. Overall, there was little Fe mobilized (Table S2). Sb(III) was not detected in R_MnR_ and was found only sporadically in R_CTRL_ at concentrations < 4 µg L^−1^ (Table S3).

### Metal fractionation

The sequential extraction analysis of the initial soil revealed that around two-thirds of the total Sb was found in the residual fraction F4 (18 ± 5 mg/kg), whereas smaller shares were found in the exchangeable fraction F1 to oxidizable fraction F3 (1.9 to 2.3 mg/kg) or were non-extractable (2.8 mg/kg) (Fig. 2a). Incubation in R_MnR_ and R_CTRL_ resulted in an equivalent decrease, respectively, in exchangeable fraction F1 (0.7 ± 0.1 and 0.9 ± 0.1 mg/kg, respectively), in reducible fraction F2 (1.1 ± 0.1 and 1.1 ± 0.2 mg/kg, respectively), in oxidizable F3 (0.5 ± 0.1 and 0.7 ± 0.1 mg/kg, respectively), as well as in residual fraction F4 (11.7 ± 0.1 and 12 ± 1 mg/kg, respectively). Notably, the non-extractable Sb fraction increased in both cases (from 2.8 to 9.8 and 8.8 mg/kg, respectively), accounting for 41% and 37% of the overall Sb left after reactor operation (Fig. 2a). Regarding Mn in the initial soil, the highest fraction of Mn was found in the reducible F2 fraction (611 ± 50 mg/kg; 48.7 ± 4.0% of total), whereas Fe was primarily (30,948 ± 3298 mg/kg; 73.7 ± 7.8% of total) found in the residual fraction F4 (Fig. 2b). The incubation in both R_MnR_ and R_CTRL_ led to a decrease in the reducible fraction F2-associated Mn fraction, whereas the concentration in the exchangeable fraction F1 fraction even increased slightly (from 86 ± 10 to 144 ± 6 and 135 ± 4 mg/kg, respectively) (Fig. 2b). The incubation had no significant effect on the Fe partitioning, i.e., the respective fractions remained virtually the same (Fig. 2c).

### Bacterial community composition and diversity

**Fig. 4:**
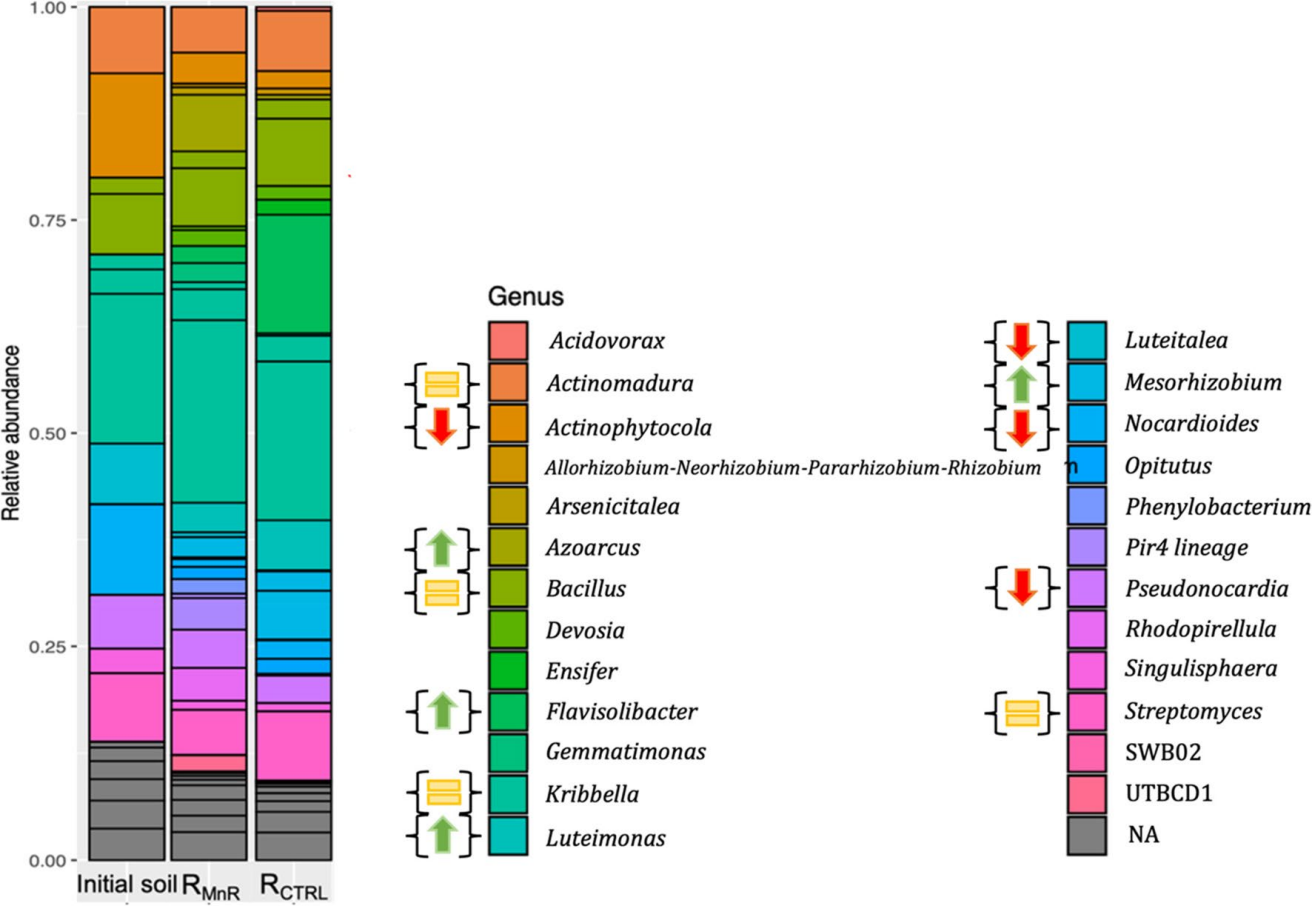
Relative abundance of genera with more than 1% abundance of all sequences in the initial soil and the soils after treatment in R_MnR_ and R_CTRL_. Arrows up mark genera that increased by more than 3% relative abundance during the incubation, arrows down indicate genera that decreased by 3%, and the equal sign represents genera that underwent no significant change of the incubated soil after 3 months of incubation, in comparison with the initial soil

The bacterial community composition in the initial soil before and after incubation in R_MnR_ and R_CTRL_ was determined at the genus level, comparing the relative abundance only of genera with more than 1% relative abundance of all sequences (Fig. 4). A total of 25 different genera were identified in the soil samples. The most abundant genera in the initial soil were *Kribbella* (Actinomycetota) and *Nocardioides* (Actinobacteriota). After the incubation period, the dominant genera were *Kribbella* (Actinomycetota), *Azoarcus* (Proteobacteria), and *Flavisobacter* (Bacteriota). The genera *Kribbella* (Actinomycetota), *Bacillus* (Firmicutes), *Actinomadura* (Actinobacteriota), and *Streptomyces* (Actinomycetota) were identified across all the soil samples accounting for 12%, 5%, 4%, and 5% of sequences in the initial soil, respectively. The genera *Nocardioides*, *Luteitalea*, and *Actinophytocola*, though present in the initial soil, were not detectable anymore after treatment, both in R_CTRL_ and R_MnR_. In contrast, the relative abundance of *Azoarcus* (*Proteobacteria*), initially present at a low number, increased to 4% in R_MnR_. Similarly, *Flavisobacter* (Bacteriota), *Luteimonas* (Proteobacteria), and *Mesorhizobium* (Proteobacteria) were not detected in the initial soil, but were found at relatively high abundances after 3 months of incubation (8%, 3%, and 3% respectively).

## Discussion

The primary objective of this study was to quantify the extent of Sb mobilization under moderately reducing conditions, compared to mobilization under more reducing conditions. The utilization of a redox-stat reactor allowed us to study processes within a fixed redox window, in an isolated manner (i.e., suppressing any other redox reaction that are thermodynamically favored at lower redox potential) for a long term (i.e., for several months). More specifically, the deliberate selection of relatively high stable redox potential in R_MnR_ (422 ± 7 mV throughout the 3-month operation; Fig. 1) thermodynamically favored the reductive dissolution of Mn while it did not favor the reductive dissolution of Fe nor Sb in R_MnR_ (see Fig. S1). Sb was exclusively released as Sb(V) (2.6 ± 0.2 mg kg^−1^ soil) in R_MnR_, whereas Sb(III) in R_CTRL_ was detected on four occasions only. Here, Sb(III) contributed to 9.6–100% to the total Sb effluent concentrations. Fe(II) was found in R_CTRL_ exclusively. Overall, little Fe was mobilized in R_CTRL_, which was in agreement with the sequential extraction results, which revealed only little Fe bound in the reducible F2 fraction (Fig. 2c). In summary, these results demonstrate that Sb reduction and Fe reduction can in principle occur in the soils studied, but both reactions were effectively suppressed at the set redox potential in R_MnR_.

Dissolved Mn was found in the effluents of R_MnR_ and R_CTRL_, respectively, throughout the whole reactor operation, suggesting that Mn(IV) and/or Mn(III) mineral oxyhydroxide dissolution was the predominant process modulating Sb release in both reactors over 80 days. The multiple linear regression analysis allowed us to describe the variance observed in Sb effluent concentration for most sampling times based on the observed Mn solubilization (Fig. 1a). In both R_MnR_ and R_CTRL_, Mn(II) concentrations represented the most significant independent variable correlating with Sb(V) concentrations, and, in fact, it was the only significant independent variable in the case of R_MnR_ (Table S4). In R_CTRL_, the regression analysis also revealed a positive correlation of Sb concentrations with pH (i.e., the higher the pH, the higher the predicted Sb effluent concentration). This is in line with increased desorption of anionic Sb(V) from negatively charged solid soil phases and may explain the slightly higher overall Sb mobilization in R_CTRL_. Notably, our regression analysis also revealed that the correlation with Pb effluent concentrations was insignificant in both reactors, indicating a preferential weathering and release of Sb from the original Pb-Sb alloy.

Interestingly, in R_CTRL_, Sb-reducing conditions were thermodynamically favored towards the end of operation, yet, nevertheless, only low levels of Sb(III) were detected. This may possibly be explained by Sb(III) sorption to Fe oxyhydroxides (Leuz et al. 2006b; Hockmann et al. 2014a, b; Liu et al. 2015). An alternative explanation could be the lack of an active Sb(V)-reducing community. Indeed, the overall microbial diversity, expressed as alpha diversity and Shannon index (Table S5), was low in contrast to a soil from a previous study, where a strong microbial diversity was observed. Enrichment attempts from the soil of this study were unsuccessful, suggesting that the lack of Sb(V) reduction in R_CTRL_ may be due to the lack of the respective community of Sb-reducing microorganisms (Figure S3).

It is well known that so-called soil aging can result in strong sequestration within soil aggregates, organic matter, or through the formation of secondary minerals resulting in the immobilization of trace elements in soils (Udovic and Lestan 2009). Still, for some soils, trace elements may be released long after industrial/contaminating activities have ceased (Rajpert et al. 2016; King et al. 2019; Verbeeck et al. 2021), in particular when redox conditions change. Here, we assessed the potential of Sb mobilization from a shooting range soil, where the last shooting activities took place 20 years ago. The shooting range soil investigated here was obtained from an environmentally unperturbed area, without direct influence from flowing water sources (e.g., by nearby streams) (BSB + Partner, Ingenieure und Planer 2007). However, the site may be susceptible to periodic waterlogging, which leads to redox oscillations, driven by fluctuations in the water level resulting from variations in precipitation. Consequently, these fluctuations impact the redox cycling of elements such as carbon (C), nitrogen (N), Fe, and Mn, ultimately influencing the biogeochemical cycling of soil contaminants, such as Sb, associated with Mn and Fe oxyhydroxides mineral structures (Bongoua-Devisme et al. 2013). Recent research has suggested that soil aging has resulted in reduced Sb toxicity and bioavailability (Verbeeck et al. 2021). Nevertheless, within the 3-month operation period in the bioreactor, a total mobilization of 10% Sb was observed both in R_MnR_ and R_CTRL_, despite the long aging period of the contaminated soil prior to its sampling in the field (Fig. 1a). This underscores the potential for long-lasting legacy effects due to the release of Sb in the environment, decades after shooting range activities have ceased. Following Swiss legislation, remediation is only necessary if the groundwater in the direct downstream of the site exceeds 10 mg L^−1^ (The Swiss Federal Council 2017). In our study, most effluent concentrations were below 10 mg L^−1^ (dashed line in Fig. 1a), exceeding neither the European drinking water maximum limit of 10 mg L^−1^ (European Union 2023), nor the maximum and recommended values in drinking water set by the World Health Organization (WHO 2018), which are 50 mg L^−1^ and 20 mg L^−1^, respectively.

Commonly, sequential extraction schemes are used to assess possible environmental fates and mobility of metals and metalloids over longer terms (Tessier et al. 1979; Quevauviller et al. 2006). In this regard, one may have expected to observe a depletion in both the exchangeable (F1) and reducible (F2) fractions of Sb since moderately and more reducing conditions were prevailing in the respective bioreactors for a long period of ~ 80 days (Fig. 1e). Despite some decrease during the incubation in the bioreactors, there was still Sb associated with F1 (2.9 and 3.9% of total Sb in R_MnR_ and R_CTRL_, resp.) and F2 (4.6% of total Sb in both cases) after bioreactor operation. Furthermore, the amount of Mn present in the “exchangeable” fraction F1 increased during treatment (to 14.5% and 16.5% of the total Mn in R_MnR_ and R_CTRL_, respectively). This may be explained by Mn(II) sorbing to soil solid phases. Nevertheless, the “reducible” F2 fraction contained most Mn (43.0% and 37.4% of the total Mn in R_MnR_ and R_CTRL_, respectively) (Fig. 2b). This suggests that Sb mobilization by reductive Mn dissolution would have continued for a relatively long time. More precisely, assuming a similar ongoing rate of relative Sb mobilization (corresponding to ~ 0.1% per day as observed in both reactors in the first 82 days of operation), one may expect Sb to be released with the effluent for another ~ 75 to 85 days until Sb fractions F1 and F2 become fully depleted of Sb (i.e., mobilized). This extrapolated time frame (weeks) is considerably shorter than time that had past after shooting activities has ceased (decades) in the field, leaving the question why there was still Sb mobilizable after all in the laboratory. We assume this is due to the experimental setup simulating continuously reducing conditions and continuous water flow through the soil. In the field, waterlogging conditions often occur temporarily, indicated visually by “redoximorphic” features. These are characteristic colorations generated by reduction, translocation, and re-oxidation of Fe and Mn phases (2018) upon fluctuating water tables. Thus, Sb mobilized through reductive dissolution in the first can (co) precipitate again when oxidizing conditions occur. In this regard, we propose redox-stat bioreactors as valuable tools to study the overall Sb mobilization potential over relatively short time scales (weeks to months), whereas field rates can only be assessed in close(r) to field setups (such as a lysimeter).

Interestingly, in both R_MnR_ and R_CTRL_, the relative fraction of Sb non-extractable by sequential extraction increased considerably (from 10.6 to 41.4% and 37.4%, respectively). Admittedly, the formation of such “residues” may be an artifact and related to the formation of insoluble Sb(V)-silicate complexes in the presence of oxidizing acids as applied during the sequential extraction (Nash et al. 2000). Possibly, it may be explained by the formation of insoluble secondary precipitates during incubation such as Sb oxides (these were suppressed in thermodynamic modeling; see supplementary information). If occurring during incubation, this may also limit the amount of Sb that can ultimately be mobilized in a natural environment.

**Fig. 5:**
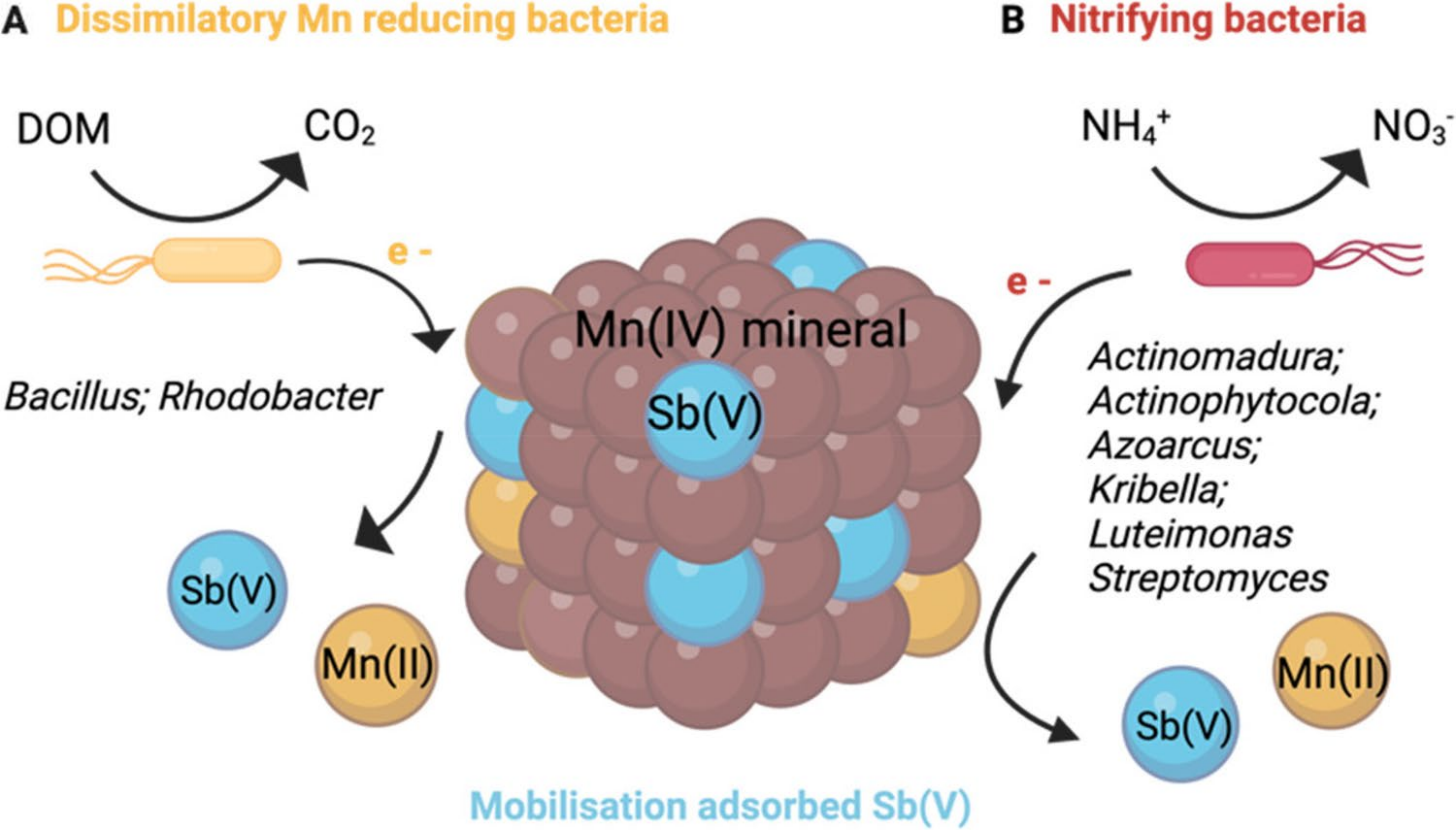
Possible electron donor mechanisms for Mn(IV) reductive dissolution based on a “conventional” understanding where DOM serves as electron donor (**A**) and a hypothetical alternative (indicated by metagenomic sequencing soil genera) where nitrification is coupled with Mn reduction (**B**). Figure created with BioRender.com

The dynamics of Mn effluent concentrations exhibited similarities between the two reactors, yet still, there was an overall 10% greater Mn mobilization observed in R_CTRL_ compared with R_MnR_, resulting in a total of 37.5% Mn mobilized (Fig. 1b). This raises the question what limits Mn reduction in R_MnR_ and/or enhances Mn reduction in R_CTRL_. This disparity may be attributed to observed differences in the microbial community during incubation. Firstly, the somewhat lower redox conditions in R_CTRL_ were conducive to the enrichment of Mn(IV)-reducing microorganisms, at least of those that are obligate anaerobes (Lovley 1993; Coates et al. 1995). Potential candidates possibly performing Mn(IV) reduction include *Bacillus infernus*, *Bacillus subterraneous*, and *Bacillus* sp. *FMR* (Boone et al. 1995; Kanso et al. 2002; Zhao et al. 2022). Indeed, the genus *Bacillus* belonged to the main genera observed, but did not display any higher relative abundance in R_CTRL_ compared to R_MnR_ (Fig. 4). Secondly, the availability of electrons for Mn reduction may have been greater in R_CTRL_ compared to R_MnR_ (reflected by the lower redox potential measured). Dissimilatory metal-reducing organisms can use different organic molecules within the DOM pool as electron donor (Fujii et al. 2010; Y. Li & Gong 2021; Gao et al. 2022) (Fig. 5). The main difference in the cumulative Mn mobilization was primarily attributable to differences in R_CTRL_ and R_MnR_ effluent concentrations observed in the time window between ~ 800 h and > 1600 h of operation (Fig. 1b). However, DOC effluent concentrations were similar in both reactors during this period. Also, the high DOC concentrations (between ~ 1000 and 2000 mg L^−1^, in both reactors) (Fig. 1f) in comparison to Mn (present in µg L^−1^; Fig. 1b) speak against any DOC electron donor limitation in R_MnR_. Lastly, one may hypothesize that there are alternative electron donors driving higher Mn reduction rates in R_CTRL_ with respect to R_MnR_. Indeed, upon examination of the metagenomic data, we observed that some of the identified genera in the incubated soils belong to bacteria known to be involved in nitrification (Fig. 4). These genera include *Actinomadura* (Lipski & Altendorf 1995; Lin et al. 2022), *Actinophytocola*, (Zhang et al. 2014; Liu et al. 2023a), *Azoarcus* (B. Liu et al. 2006; Lee et al. 2014; Li et al. 2020), *Luteitalea* (Pessi et al. 2022), *Mesorhizobium* (Siddiqi et al. 2019), and *Streptomyces* (Feng et al. 2014; He et al. 2021, 2022). Under the assumption that these bacterial communities were active in R_CTRL_ and ammonia (NH_4_^−^) was present (likely due to animal excreta and/or decaying organic matter), nitrification may have been coupled to Mn reduction (Fig. 5b). The anoxic bio-oxidation pathway of NH_4_^−^ in the presence of MnOx was first observed by Hulth et al. (1999). Microbial-mediated manganese redox cycling for the simultaneous removal of NO_3_^−^/NO_2_^−^ and NH_4_^−^, along with micropollutants, has recently gained growing interest (Liu et al. 2023a, b; Zhong et al. 2023). However, since we did not quantify nitrogen species during reactor operation, this remains a hypothesis, the testing of which will require further research.

In this study, we provide clear evidence that the transition from oxic to moderately reducing conditions can mobilize substantial quantities of Sb in contaminated shooting range soils, even decades after shooting activities have ceased. Our findings reveal that this mobilization is closely coupled to the reductive dissolution of Mn mineral phases, which otherwise help to retain the Sb in soils. Hence, this study underscores that in addition to the well-known control Fe mineral oxyhydroxides exert on trace elements in soils, Mn oxyhydroxides can similarly contribute to trace metal mobilization, despite their generally lower concentration in the environment in comparison to Fe. Since Mn oxyhydroxides already dissolve under moderately reducing conditions, such redox-active mineral phases may play even the dominant role in the upper layers of waterlogged soils facing only suboxic conditions. While the redox potential and reductive Mn dissolution are certainly important constraints on, or even predictors of, Sb mobility, and robust relationships between Mn and Sb release may be observed for a given environment, such relationship likely vary with environmental conditions (e.g., redox conditions, microbial community structure, the soil chemistry in Si or Ca rich soils) and with the contribution of Mn-independent microbial Sb reduction. Here, we did not observe much microbial Sb reduction even though this reaction should be thermodynamically favored. Our study thus underscores the need towards a better understanding of the microbial communities within Sb-contaminated soils, exerting a key role as biocatalysts conferring reductive reactions. We recommend further microbial community analysis over time, and in different contaminated sites, as well as transcriptomic analyses (specifically targeting Sb-reducing genes) to gain a comprehensive understanding of key microbial taxa and proteins that contribute directly or indirectly to Sb mobilization within Sb-contaminated environments.

## Supplementary Information

Below is the link to the electronic supplementary material.

Supplementary file1 (PDF 678 KB)

## Data Availability

The authors declare that data supporting the findings of this study is available within the Supplementary Information. Additionally, any raw data files are available from the corresponding author upon reasonable request.
