# Supplementary material for: Manganese reductive dissolution coupled to Sb mobilization in contaminated shooting range soil
Source: Appl Microbiol Biotechnol. 2024 Apr 10;108:295. doi: 10.1007/s00253-024-13133-2 (PMC11006745; doi:10.1007/s00253-024-13133-2)
Supplement: Supplementary file 1 [file 253_2024_13133_MOESM1_ESM.pdf]

Supplementary Information: Manganese reductive dissolution coupled to Sb mobilisation in contaminated shooting range soil

Lara Costa [1,2], Mathieu Martinez [1], Marcel Suleiman [1], Rolf Keiser [3], Moritz Lehmann [2], Markus Lenz [1, 4]

[1] Institute for Ecopreneurship, School of Life Science, University of Applied Sciences and Arts Northwestern Switzerland (FHNW); Hofackerstrasse 30, 4132 Muttenz, Switzerland
[2] University of Basel, Department of Environmental Science; Bernoullistrasse 30, 4056 Basel, Switzerland
[3] ARMASUISSE Competence Center Soil; Guisanplatz 1, 3003 Bern, Switzerland
[4] Sub-Department of Environmental Technology, Wageningen University; 6700 EV Wageningen, The Netherlands

Elemental Analysis

Table S1 Elemental concentrations determined by XRF in comparison to certified values in reference material "BCR-176R fly ash".

	Sb	Fe	Mn
**Certified value [mg kg^{-1}]**	850 ± 50	13100 ± 500	730 ± 50
**Determined vale [mg kg^{-1}]**	784 ± 4	14060 ± 20	855 ± 8

Table S2 Total Fe, Fe^{2+} Fe^{3+} concentrations in mg kg^{-1} mobilized from soil throughout 82 days of reactor operation.

	Fe^{3+}	Fe^{2+}	Fe total
**R_{MnR}**	33.8 ± 3.9	0.0 ± 0.0	33.8 ± 3.9
**R_{CTRL}**	37.4 ± 4.2	6.4 ± 1.1	43.7± 5.2

Sb(III) was only sporadically detected during the operation of RCTRL: 3.3 μgL^{-1} (770 h); 3.9 μgL^{-1} (818 h); 0.5 μgL^{-1} (866 h) and 0.63 μgL^{-1} (1250 h).

Table S3 Elemental concentrations quantified by X-ray fluorescence (XRF) spectroscopy of the initial soil ($C_{initial}$), and the final concentrations (C_{final}) after incubation in R_{MnR} and R_{CTRL}, respectively. The experimental recovery (in %) was calculated as the sum of C_{final} and the cumulative concentration mobilized during reactor operation, divided by the respective $C_{initial}$.

	R_{CTRL}				R_{MnR}		
	**C total initial [mg kg^{-1}]**	**C total final [mg kg^{-1}]**	**C mobilised by XRF [mg kg^{-1}]**	**Exp. recovery [%]**	**C total final [mg kg^{-1}]**	**C mobilised by XRF [mg kg^{-1}]**	**Exp. recovery [%]**
**Sb**	27 ± 4	23 ± 3	2 ± 1	97.03	24 ± 3	2 ± 1	98.40
**Mn**	1254 ± 49	814 ± 5	441 ± 52	103.00	992 ± 13	263 ± 60	107.00
**Fe**	42018 ± 535	40507 ± 183	1727 ± 524	96.50	40850 ± 803	1448 ± 218	97.30

Thermodynamic modelling

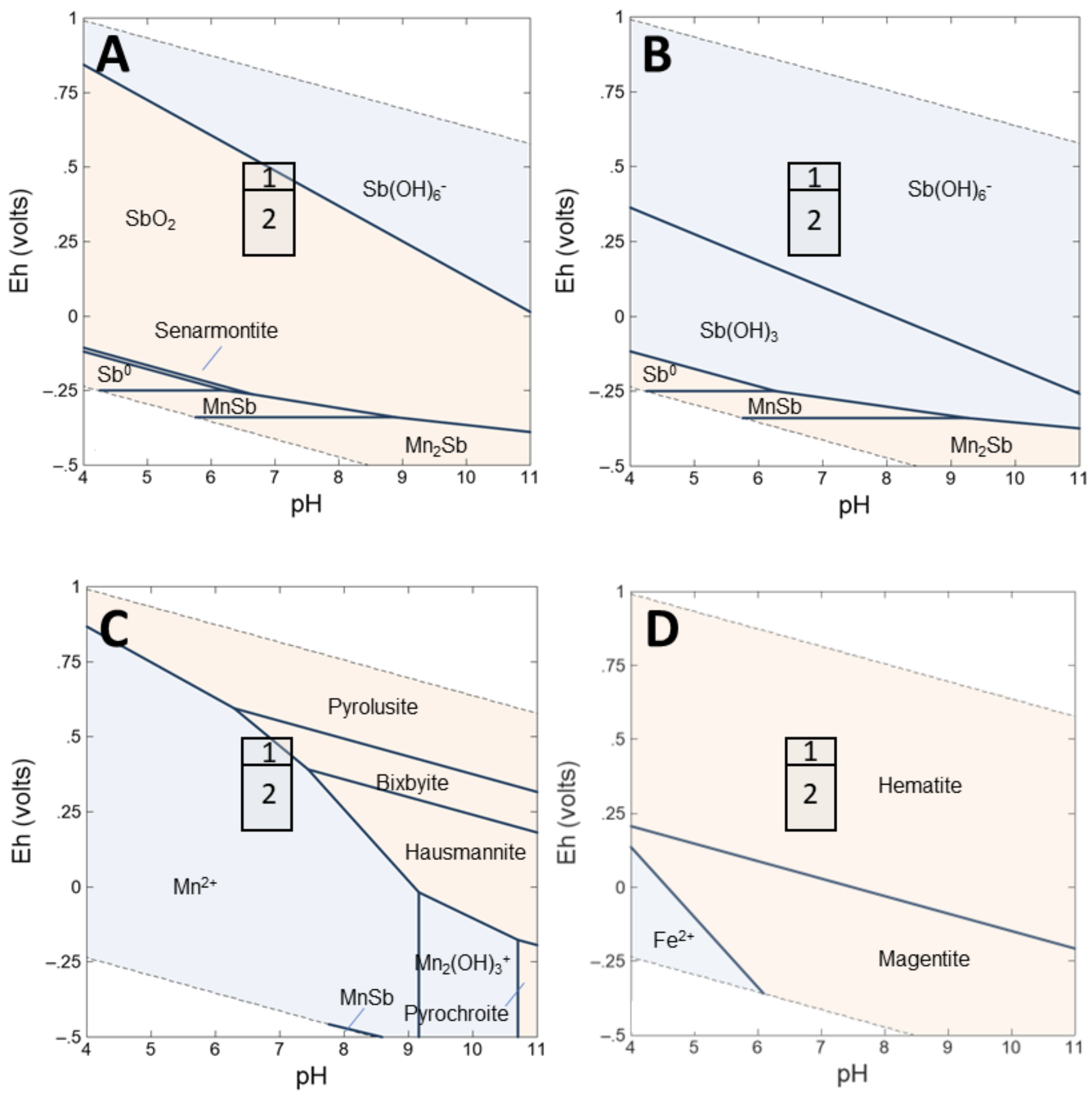

Fig. S1 Pourbaix diagrams generated using the MINTEQ database in Geochemist Workbench (GWB) for a 10% (w/w) slurry of the initial soil in pure water. Sb (a, b) Mn (c) and Fe (d) at 25°C. The blue areas represent dissolved species, whereas the orange areas represent solids. The boxes indicate the range of pH and Eh measured in R_{MnR} (1) and R_{CTRL} (2). Note that SbO_2 was suppressed for modelling.

The thermodynamic model was based on the concentrations of Sb, Fe and Mn of the initial soil, assuming that 100g was suspended in pure water, not containing any ions. For Sb, a full model is shown in **Figure S1a**, containing all species of the Minteq database. Since some of the Sb oxides require high temperatures for synthesis (see e.g. (Chin et al., 2010) and / or occur rarely in nature (e.g. senarmontite, Sb_2O_3; https://www.mindat.org/min-3618.html) they were suppressed in **Figure S1b**. As a result, $Sb(OH)_3$ becomes visible, which was in fact measured in R_{CTRL} (details see main manuscript). The effluent redox potential read out (x) of the electrode was converted to E_h using the following formula, ($E_{0(21°C)}$ = **x + 219mV**), assuming a 3M KCl system (https://www.wolkersdorfer.info/en/redoxprobes.html) and added manually to Figure S1.

Bioreactors set-up

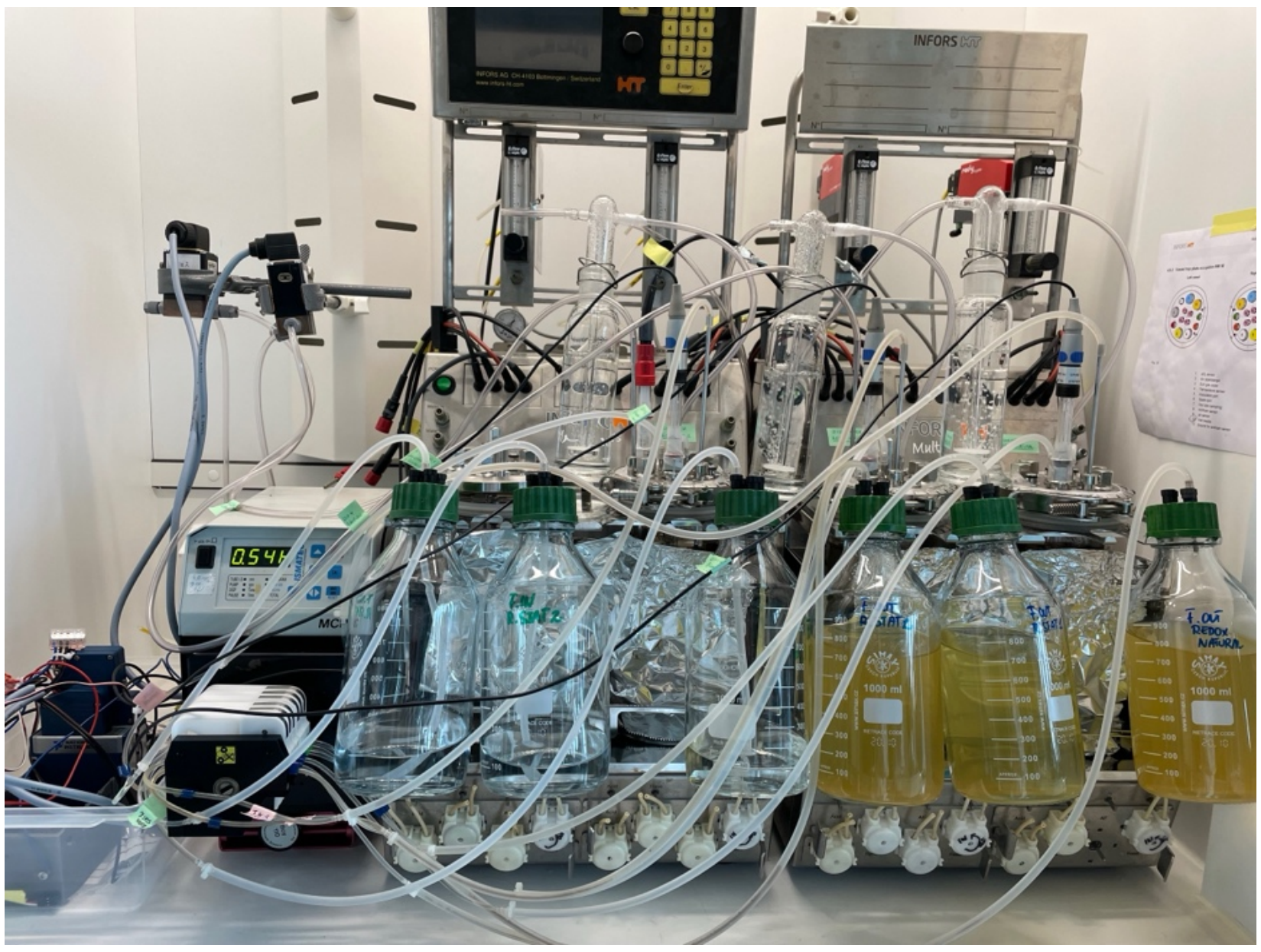

Fig. S2 Reactors set-up system.

Multiple linear regression analysis

Table S4 Multiple linear regression analysis using multiple variables for R_{MnR} (coefficient of determination, $R^2 = 0.44$) and R_{CTRL}, ($R^2 = 0.48$). The asterisk represents the variables with statistical significance ($p < 0.05$).

	R_{CTRL}				R_{MnR}			
	**Coef.**	**Standard Error**	**t Stat**	**P-value**	**Coef.**	**Standard Error**	**t Stat**	**P-value**
**Intercept**	-53	21.3	2.49	**0.0174***	-33	22.6	1.46	0.1511
**Redox [mV]**	0.0202	0.0061	3.33	**0.0019***	0.0169	0.0190	0.89	0.3800
**pH**	7	2.88	2.43	**0.0201***	4.25	2.37	1.80	0.0798
**Mn [µg L⁻¹]**	0.0034	0.0006	5.78	**<0.0001***	0.0023	0.0004	5.28	**<0.0001***
**Pb [µg L⁻¹]**	-0.0431	0.0295	1.46	0.1514	0.0211	0.0318	0.66	0.5115
**Fe [µg L⁻¹]**	-0.0013	0.0063	0.02	0.8335	0.0046	0.0066	0.69	0.4917
**DOC [mg L⁻¹]**	0.0007	0.0006	1.10	0.2785	-0.0004	0.0004	-0.89	0.3766

The predicted values for Sb concentrations in R_{CTRL} and R_{MnR} (see Fig. 3 in main manuscript) were calculated based on **Equation S1** and **Equation S2**, respectively.

$$\boldsymbol{Sb_{effluent\ RCTRL}}\ [\boldsymbol{mg\ L^{-1}}] = -53 + 0.0202\ \times redox\ [mV] + 7 \times pH + 0.0034\ \times Mn[mg\ L^{-1}] - 0.0431 \times\ Pb[mg\ L^{-1}]\ - 0.0013\ \times\ Fe[mg\ L^{-1}] + 0.0007\ \times\ DOC\ [mg\ L^{-1}]$$

Equation 1

$$\boldsymbol{Sb_{effluent\ RMnR}}\ [\boldsymbol{mg\ L^{-1}}] = -33 + 0.0169\ \times redox\ [mV] + 4.25 \times pH + 0.0023\ \times Mn[mg\ L^{-1}] - 0.0211\ \times\ Pb[mg\ L^{-1}] + 0.0046\ \times\ Fe[mg\ L^{-1}] - 0.0004\ \times\ DOC\ [mg\ L^{-1}]$$

Equation 2

Calculation of trace metal mobilisation rates and cumulative trace metal mobilisation

Based on the total dissolved effluent concentrations and the reactor design, the trace metal mobilisation rates (TMR; μg L^{-1} h^{-1}) and cumulative trace metal mobilised ($TM_{cumulative}$; μg g^{-1} soil) were calculated according to **Equation S3** and **Equation S4**, respectively.

$$TMR = \frac{C_{effuent} \times Q}{V}$$

Equation 3

$$TM_{cumulative} = \sum_{i=1}^{n} \left[\frac{TMR_{i+1} \times V \times (t_{i+1} - t_i)}{wt} \right]$$

Equation 4

Where $C_{effluent}$ is the effluent concentration [mg L^{-1}], Q is the flow rate [L h^{-1}], and V is the reactor volume [L]. T is the operation time [h] and wt represents the initial soil weight[g].
The $TM_{cumulative}$ [%] was determined by dividing $TM_{cumulative}$ by the initial concentration of the trace metal (mg g^{-1} soil) in the soil.

Microbial diversity of soils from Brocheten (this study) and Chur (Hockmann et al., 2014)

Table S5 Alpha-diversity and Shannon index of the study soil and the "Chur" soil used by (Hockmann et al., 2014).

	Alpha	Shannon
**Brocheten soil**	304	4.07
**Chur soil**	1434	6.73

Soil enrichment cultures

Growth/enrichment experiments were performed to test the dissimilatory Sb-reduction potential/capacity of the soils. The soil characterised in this work (Brocheten) and the soil *Chur* described previously (Hockmann, 2014) were used for the enrichments. Chur soil was used as a point of reference, as previous work showed that the soil hosts an active Sb (and Fe) reducing microbial community, and efficient dissimilatory Sb(V) reduction to Sb(III) was clearly demonstrated under the experimental conditions applied also in this study. The media used was the basal salt medium (BSM) containing (in gL^{-1} of deionised water) 0.240 $K_2CO_3{\cdot}1.5H_2O$; 0.1 KH_2PO_4; 0.15 K_2HPO_4, 10 mL vitamin solution and 1 mL SL-10 trace metal solution supplemented with 14 mM $MgCl_2$ and 700 mM $CaCl_2$. The BSM was sterilised by autoclaving (121 °C for 45 min), and the vitamin and trace solutions were previously filtered through 0.45 mm PVDF filters. All solutions were made anoxic prior to use, by purging with N_2. For the bacterial enrichment, 1 g (wet weight) of soil in 100 mL BSM was added to 500 mL sterile serum bottles, and amended with

1 mM $KSb(OH)_6$ (TEA, terminal electron acceptor) and with 2 mM sodium L-lactate (carbon source and electron donor). The $KSb(OH)_6$ and sodium L-lactate solutions were prepared using filtered de-ionised water and were purged with N_2 to remove the oxygen. After adding the soil and medium, the serum bottles were closed with sterilised butyl rubber stoppers, crimp-sealed with aluminium caps, and placed in the dark on a rotatory shaker at 30°C. Four sets of duplicates were incubated: soil incubated in BSM media supplemented with 1mM $KSb(OH)_6$ and 2 mM sodium L-lactate, soil incubated in BSM with 1mM $KSb(OH)_6$, soil incubated in BSM with 2 mM sodium L-lactate (biotic control lacking an added TEA) and BSM supplemented with 1mM $KSb(OH)_6$, and 2 mM sodium L-lactate without soil (abiotic control). Samples for analysis of Sb speciation [Sb(V)/Sb(III) concentrations] were collected weekly from the reactors, using a sterile needle syringe. All manipulations were conducted inside an anoxic glove box to prevent O_2 contamination.

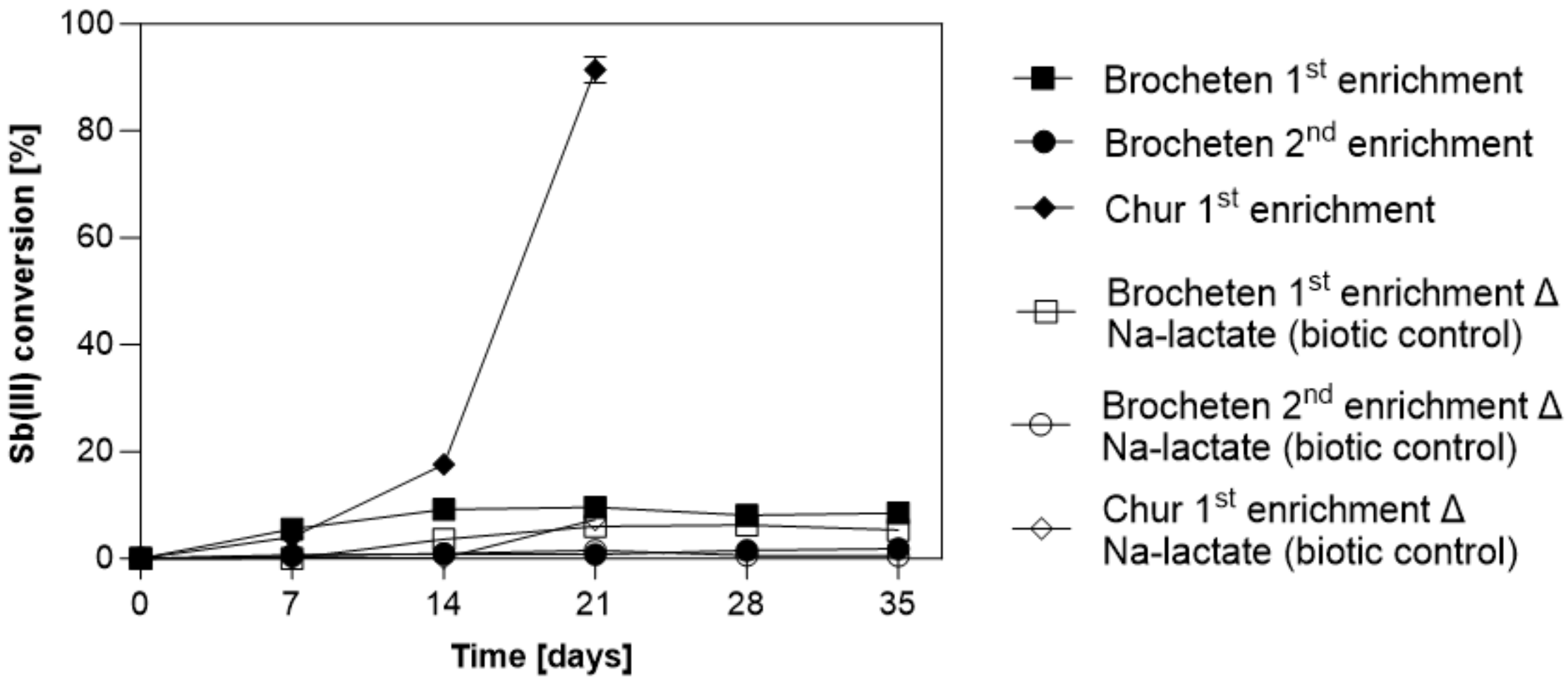

Fig. S3 Conversion of Sb(V) to Sb(III) [%] during a series of enrichments with the Brocheten soil and Chur soil incubated under anoxic conditions.

The results of the enrichment with the soil of this study showed a conversion of Sb(V) to Sb(III) of 7.62 ± 0.06% after 35 days, while the enrichment with the *Chur* soil previously used showed a conversion of 91.46 ± 2.44% already within 21 days (**Figure S2**). A second enrichment experiment with *Brocheten* soil qualitatively confirmed the initial results, but the overall fractional Sb(V) reduction was even lower, with only 1.85 ± 0.01% (**Figure S2**). The *Brocheten* and *Chur* soils amended only with $KSb(OH)_6$ and without Na-lactate (biotic controls) revealed a reductive Sb(V) conversion of 8.12 ± 0.12% and 7.28 ± 1.76%, respectively. These findings indicate that natural organic matter (NOM) can, to some extent, serve as electron donor and C source (in the case of *Chur* enrichments). It is not clear, what exactly explains the differential Sb-reduction potential between the two tested soils, and we suspect that, in the absence of a larger Sb-reducing microbial community, part of the slow/low observed Sb(V)->Sb(III) conversion may be attributed to abiotic processes. In any case, unlike in the *Chur* soil, microbially mediated dissimilatory Sb reduction seems to play a subordinate role in the *Brocheten* soils used in our reactor incubations.
